# Supplementary material for: A green lifetime biosensor for calcium that remains bright over its full dynamic range
Source: eLife. 2025 Dec 19;14:RP105086. doi: 10.7554/eLife.105086 (PMC12716836; doi:10.7554/eLife.105086)
Supplement: Supplementary file 1. [file elife-105086-supp1.docx]

Sensor variants are indicated by their circular permutation and mutations in the FP in the sensor. For example, ‘cp146’ has from N- to C-terminus the following domains: calmodulin binding peptide M13, amino acids 146-283 of mTq2, a flexible GGSGG linker, amino acids 1-145 of mTq2, CaM (calmodulin). ‘T203Y’ indicates a T203Y mutation on the mTq2. The calcium-bound state is measured in presence of 0.1 mM CaCl_2_ and the calcium-free state after addition of 9.5 mM EDTA.

*Emission and excitation maxima are only indicated if a red shift of the spectrum with respect to Tq-Ca-FLITS was observed.

**Phase lifetimes were measured at 37 °C by FD-FLIM. The lifetime change is calculated as the phase lifetime in the calcium-bound state minus the calcium-free state.

| **Sensor variant** | **Emission maximum (nm)*** | | **Excitation maximum (nm)*** | | **Phase lifetime (ns) **** | | |
| --- | --- | --- | --- | --- | --- | --- | --- |
|  | **calcium free** | **calcium bound** | **calcium free** | **calcium bound** | **calcium free** | **calcium bound** | **change** |
| **Tq-Ca-FLITS** | 492, 502 | 480, 507 | 437, 455 | 437, 456 | 1.72 | 2.86 | 1.14 |
| **T203Y** | 516 | 514 | 461, 488 | 459, 478 | 3.08 | 2.20 | -0.88 |
| **cp146, T203Y** | 518 | 517 | 461, 480 | 460, 479 | 3.40 | 3.13 | -0.28 |
| **cp147, T203Y** | 517 | 518 | 459, 480 | 460, 481 | 3.02 | 3.38 | 0.36 |
| **cp149, T203Y** | 516 | 518 | 461, 481 | 460, 479 | 2.69 | 2.65 | -0.04 |
| **cp150, T203Y** | 512, shoulder 490 | 515 | 460, shoulder 435 | 460, 478 | 1.85 | 2.37 | 0.52 |
| **cp151, T203Y** | 516 | 516 | 460, 479 | 460, 479 | 2.73 | 2.91 | 0.18 |
| **cp152, T203Y** | 516 | 517 | 460, 480 | 460, 479 | 3.16 | 3.05 | -0.11 |
| **cp146, T203H** | no red shift | no red shift | no red shift | no red shift | 3.09 | 3.21 | 0.12 |
| **cp147, T203H** | no red shift | no red shift | no red shift | no red shift | 3.13 | 2.86 | -0.27 |
| **cp149, T203H** | no red shift | no red shift | no red shift | no red shift | 2.67 | 2.78 | 0.11 |
| **cp150, T203H** | no red shift | no red shift | no red shift | no red shift | 2.60 | 2.96 | 0.36 |
| **cp152, T203H** | no red shift | no red shift | no red shift | no red shift | 3.15 | 3.01 | -0.13 |
