# Supplementary material for: A green lifetime biosensor for calcium that remains bright over its full dynamic range
Source: eLife. 2025 Dec 19;14:RP105086. doi: 10.7554/eLife.105086 (PMC12716836; doi:10.7554/eLife.105086)
Supplement: Supplementary file 2. [file elife-105086-supp2.docx]

| No. | Sequence | Internal name |
| --- | --- | --- |
| *Engineering G-Ca-FLITS* | |  |
| 1 | CAACCACTACCTGAGCYACCAGTCCAAGCTGAGCAAAGAC | FW_Tq2_T203Y/H |
| 2 | GTRGCTCAGGTAGTGGTTGTCGG | RV_Tq2_T203Y/H |
| 3 | CAACCACTACCTGAGCTTCCAGTCCAAGCTGAGCAAAGAC | FW_Tq2_T203F |
| 4 | GAAGCTCAGGTAGTGGTTGTCGG | RV_Tq2_T203F |
| 5 | CAAGGCCAACTTCAAGYDSCGCCACAACATCGAGGAC | Fw_Tq2_I167arom |
| 6 | SHRCTTGAAGTTGGCCTTGATGCCG | Rv_Tq2_I167arom |
| 7 | GCTGAGCTCACCCGTGNNKGTCTATATCACCGCCG | FW_G-FLITS_PVX-linker |
| 8 | GTCACGCGTMNNGTCGCTATAGTAGTTGTAC | RV_G-FLITS_SDX-end |
| *Creation dual expression ratio plasmid* | |  |
| 9 | CGGCATGGACGAGCTGTACAAGTCGACCAG | FW-del_SacI_in-FP-pDress |
| 10 | CTGGTCGACTTGTACAGCTCGTCCATGCCG | RV-del_SacI_in-FP-pDress |
| 11 | GTCGCGAATTCAGGCGC | FW_FP-lessFRET-P2A |
| 12 | TTTGGATCCAGCGCTAGCGAAGGTCC | RV_FP-lessFRET-P2A |
| 13 | TAAGGATCCCGCCACAATGGTC | FW_FLITS-backbone_notags |
| 14 | GCGCCTGAATTCGCGAC | RV_FLITS_backbone_notags |
| *Sensors and FPs in ratioplasmid* | |  |
| 15 | AACCACTACCTGAGCTACCAGTCCAAGCTGAGC | FW_Tq2_T203Y |
| 16 | GCTCAGCTTGGACTGGTAGCTCAGGTAGTGGTT | RV_Tq2_T203Y |
| 17 | GCTGGATCCCGCCACCATGGTGAGC | FW_FP_to_pFR |
| 18 | CACAAGCTTTTACTTGTACAGCTCGTCC | RV_FP_to_pFR |
| 19 | GCTGGATCCCGCCACA ATGGTCGACTCATCACG | FW_RCaMPs_to_ratio |
| 20 | CACAAGCTTCTACTTCGCTGTCATCATTTG | RV_RCaMPs_to_ratio |
| *Organelle targeting* | |  |
| 21 | GCTACCGGTCGCCACCATGGTCGACTCTTCACG | FW_VY_to_C1N1 |
| 22 | TTTTGTACACCTTCGCTGTCATCATTTGGACAAACTC | RV_VY_to_C1N1 |
| 23 | TTTTGTACACCTACTTCGCTGTCATCATTTGGACAAACTC | RV_VY-STOP_to_C1N1 |

Mixed bases are indicated by special letters: Y (C or T), R (A or G), K (G or T), M (A or C), D (A, G or T), H (A, C or T) and N (any).
